# 2-[3-(Pyridin-1-ium-2-yl)-1*H*-pyrazol-1-yl]-6-[3-(pyridin-2-yl)-1*H*-pyrazol-1-yl]pyridinium sulfate methanol monosolvate

**DOI:** 10.1107/S1600536813008647

**Published:** 2013-04-10

**Authors:** Linxia Huang, Mouhai Shu

**Affiliations:** a School of Chemistry and Chemical Engineering, State Key Laboratory of Metal Matrix Composites, Shanghai Jiao Tong University, Shanghai 200240, People’s Republic of China

## Abstract

The title solvated salt, C_21_H_17_N_7_^2+^·SO_4_^2−^·CH_3_OH, was obtained when we attempted to prepare the complex of ferrous sulfate and 2,6-bis­[3-(pyridin-2-yl)-1*H*-pyrazol-1-yl]pyridine in methanol. The dihedral angles between adjacent pyridine and pyrazole rings range from 3.8 (1) to 13.4 (1)°. An intra­molecular N—H⋯N hydrogen bond occurs. In the crystal, N—H⋯O and O—H⋯N hydrogen bonds between solvent methanol mol­ecules and the cations generate zigzag chains along [110].

## Related literature

For general background to the chemistry of oliga­pyridine ligands, see: Constable *et al.* (1988 ▶, 1992 ▶, 1997 ▶); Fu, Li *et al.* (1996 ▶); Fu, Sun *et al.* (1996 ▶). For the synthesis of the ligand, see: Jameson & Goldsby (1990 ▶).
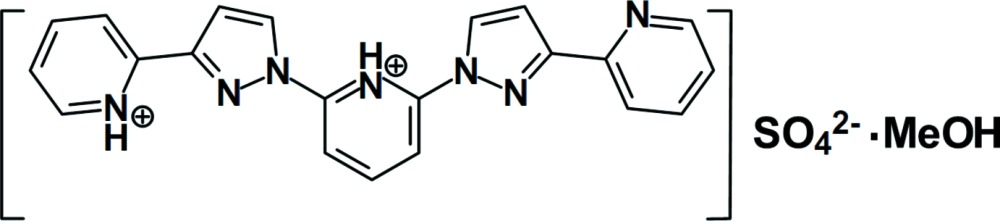


## Experimental

### 

#### Crystal data

C_21_H_17_N_7_^2+^·SO_4_^2−^·CH_4_O
*M* *_r_* = 495.52
Triclinic, 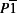

*a* = 9.2575 (5) Å
*b* = 12.1707 (7) Å
*c* = 12.1991 (7) Å
α = 112.786 (6)°
β = 100.997 (5)°
γ = 106.363 (5)°
*V* = 1143.90 (11) Å^3^
*Z* = 2
Mo *K*α radiation
μ = 0.19 mm^−1^
*T* = 293 K
0.26 × 0.23 × 0.20 mm

#### Data collection

Bruker APEX CCD area-detector diffractometer
Absorption correction: multi-scan (*SADABS*; Sheldrick, 2003 ▶) *T*_min_ = 0.952, *T*_max_ = 0.963
7326 measured reflections
4192 independent reflections
2629 reflections with *I* > 2σ(*I*)
*R*_int_ = 0.027

#### Refinement

*R*[*F* ^2^ > 2σ(*F* ^2^)] = 0.053
*wR*(*F* ^2^) = 0.163
*S* = 1.01
4192 reflections
334 parameters
21 restraints
H atoms treated by a mixture of independent and constrained refinement
Δρ_max_ = 0.28 e Å^−3^
Δρ_min_ = −0.39 e Å^−3^

### 

Data collection: *SMART* (Bruker, 2000 ▶); cell refinement: *SAINT* (Bruker, 2000 ▶); data reduction: *SAINT*; program(s) used to solve structure: *SHELXS97* (Sheldrick, 2008 ▶); program(s) used to refine structure: *SHELXL97* (Sheldrick, 2008 ▶); molecular graphics: *SHELXTL* (Sheldrick, 2008 ▶); software used to prepare material for publication: *publCIF* (Westrip, 2010 ▶).

## Supplementary Material

Crystal structure: contains datablock(s) global, I. DOI: 10.1107/S1600536813008647/kj2223sup1.cif

Structure factors: contains datablock(s) I. DOI: 10.1107/S1600536813008647/kj2223Isup2.hkl

Supplementary material file. DOI: 10.1107/S1600536813008647/kj2223Isup4.mol

Supplementary material file. DOI: 10.1107/S1600536813008647/kj2223Isup4.cml

Additional supplementary materials:  crystallographic information; 3D view; checkCIF report

## Figures and Tables

**Table 1:** Hydrogen-bond geometry (Å, °)

*D*—H⋯*A*	*D*—H	H⋯*A*	*D*⋯*A*	*D*—H⋯*A*
N7—H7⋯N6	0.86 (1)	2.35 (1)	2.713 (10)	106 (1)
O5—H5⋯N1	0.86 (3)	1.97 (3)	2.79 (3)	159 (4)
N7—H7⋯O5^i^	0.86 (1)	1.88 (1)	2.690 (10)	156 (1)

Symmetry code: (i) 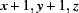.

**Fig. 1.**
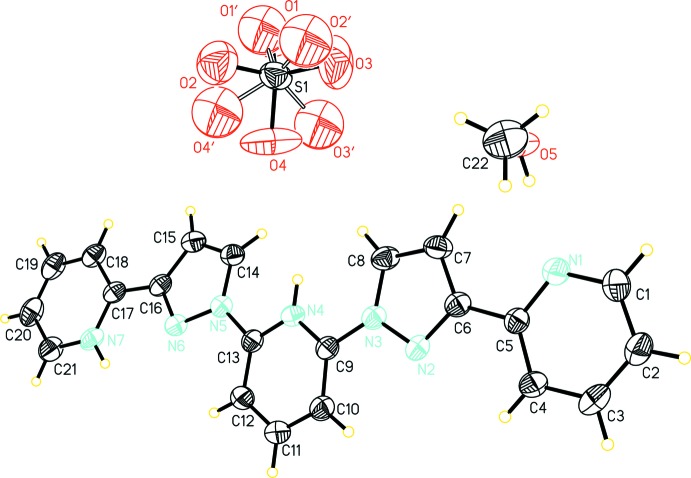
The molecular structure of the title complex with atom labels and 30% probability displacement ellipsoids for non-H atoms.

**Fig. 2.**
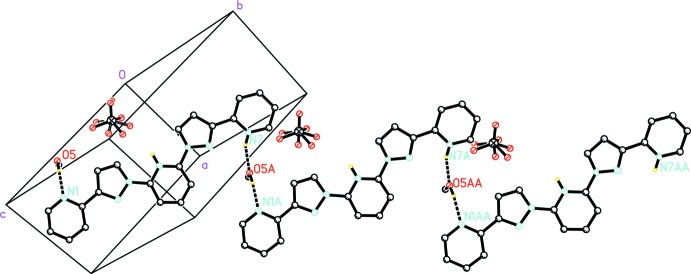
The chain formed by the lintermolecular N—H···O, and O—H···N hydrogen bonds (dashed lines) in the crystal. H atoms not involved in hydrogen bonding have been omitted for clarity.

## supplementary crystallographic information

### Comment

Helicates can be obtained by the self assembly of oligopyridine ligands with transition metal ions (Constable, 1992). 2,6':2',6":2",6'":2'",6""-Quinquepyridine reacts with Ag^I^ ions to give a mononuclear single-stranded helical complex (Constable *et al.* 1988). Mn^II^ single-stranded helicates bridged by Cl^-^ (Fu, Li *et al.* 1996) and Ag^I^ dinuclear double-stranded helicates (Fu, Sun *et al.* 1996) were obtained from the quinquepyridine when methyl groups were introduced at the 6 and 6"" positions. The presence of alkyl groups bound to the 4 and 4' positions of quaterpyridine leads to the complete formation of the head-to-head conformer over the head-to-tail conformer (Constable *et al.* 1997). This encouraged us to investigate the coordination chemistry of transition metal ions with a new ligand containing a N_5_ donor set. In this work, 2,6-di[3-(2-pyridyl)-1*H*-pyrazol-1-yl]-pyridine (Jameson & Goldsby, 1990) was used to react with ferrous sulfate in methanol, and the title compoud was obtained as yellow crystals.

In the structure, dihedral angles between the pyrazole and the pyridine rings (the rings are defined by the nitrogen atoms) are as follows: N1/N2N3 = 3.8 (1), N2N3/N4 = 4.3 (1), N4/N5N6 = 13.4 (1), N5N6/N7 =4.3 (1) °. Intramolecular N—H···N hydrogen bond and intermolecular N—H···O, and O—H···N hydrogen bonds were observed in the crystal. The intermolecular N—H···O and O—H···N hydrogen bonds between solvent methanol molecules and the organic molecules generate zigzag hydrogen bond chains running in the [110] direction.

### Experimental

2,6-Di[3-(2-pyridyl)-1*H*-pyrazol-1-yl]-pyridine was prepared using methods described in the literature (Jameson & Goldsby, 1990). A solution of 2-(1*H*-pyrazol-3-yl)-pyridine (11.76 g, 81 mmol) in 100 ml of anhydrous 2-methoxyethyl ether was stirred with potassium (6.0 g, 153 mmol) at 70 ° C under argon until the metal dissolved. To this solution was aadded 2,6-dibromopyridine (5.90 g, 24.8 mmol) in one portion. The mixture was stirred at 110 ° C for 4 days. The crude product was washed with hot water twice, and recrystallized from dichloromethane-hexane, 2,6-di[3- (2-pyridyl)-1*H*-pyrazol-1-yl]-pyridine was obtained as light yellow powder (yield 60%).

2,6-Di[3-(2-pyridyl)-1*H*-pyrazol-1-yl]-pyridine (18.3 mg, 0.05 mmol) and FeSO_4_.4H_2_O (14 mg, 0.05 mmol) were mixed in methanol (3 ml) in a vial, the vial was covered and heated to 60 ° C for 48 h. After cooling, the title compound was obtained as yellow crystals suitable for X-ray structure analysis.

### Refinement

H atoms bonded to O atoms were located in a difference map. Other H atoms were positioned geometrically and refined using a riding model with N—H = 0.86 (aromatic), C—H = 0.93 (aromatic) and C—H = 0.96 (CH_3_). All H atoms were refined with *U*_iso_(H) = 1.2 times (1.5 for methyl groups) *U*_eq_(C). The four oxygen atoms in sulfate anion are disordered over two positions. The site occupancy factors of these disordered oxygen atoms were refined by free variable to 0.782 (10) for O1, O2, O3 and O4, and 0.218 (10) for O1', O2', O3'and O4', respectively, with distances restraints of S—O = 1.44 (1) Å and angles restraints of O—S—O = 109.5°. Only the major component O atoms were refined with anisotropic displacement parameters.

### Crystal data

**Table:**

C_21_H_17_N_7_^2+^·SO_4_^2^^−^·CH_4_O	*Z* = 2
*M**_r_* = 495.52	*F*(000) = 516
Triclinic, *P*1	*D*_x_ = 1.439 Mg m^−^^3^
Hall symbol: -P 1	Mo *K*α radiation, λ = 0.71073 Å
*a* = 9.2575 (5) Å	Cell parameters from 2589 reflections
*b* = 12.1707 (7) Å	θ = 3.3–29.3°
*c* = 12.1991 (7) Å	µ = 0.19 mm^−^^1^
α = 112.786 (6)°	*T* = 293 K
β = 100.997 (5)°	Block, yellow
γ = 106.363 (5)°	0.26 × 0.23 × 0.20 mm
*V* = 1143.90 (11) Å^3^	

### Data collection

**Table:**

Bruker APEX CCD area-detector diffractometer	4192 independent reflections
Radiation source: fine-focus sealed tube	2629 reflections with *I* > 2σ(*I*)
Graphite monochromator	*R*_int_ = 0.027
Detector resolution: 10.3592 pixels mm^-1^	θ_max_ = 25.4°, θ_min_ = 3.3°
phi and ω scans	*h* = −11→11
Absorption correction: multi-scan (*SADABS*; Sheldrick, 2003)	*k* = −12→14
*T*_min_ = 0.952, *T*_max_ = 0.963	*l* = −14→14
7326 measured reflections	

### Refinement

**Table:**

Refinement on *F*^2^	Primary atom site location: structure-invariant direct methods
Least-squares matrix: full	Secondary atom site location: difference Fourier map
*R*[*F*^2^ > 2σ(*F*^2^)] = 0.053	Hydrogen site location: inferred from neighbouring sites
*wR*(*F*^2^) = 0.163	H atoms treated by a mixture of independent and constrained refinement
*S* = 1.01	*w* = 1/[σ^2^(*F*_o_^2^) + (0.092*P*)^2^ + 0.0074*P*] where *P* = (*F*_o_^2^ + 2*F*_c_^2^)/3
4192 reflections	(Δ/σ)_max_ < 0.001
334 parameters	Δρ_max_ = 0.28 e Å^−^^3^
21 restraints	Δρ_min_ = −0.39 e Å^−^^3^

### Special details

**Table:**

Geometry. All e.s.d.'s (except the e.s.d. in the dihedral angle between two l.s. planes) are estimated using the full covariance matrix. The cell e.s.d.'s are taken into account individually in the estimation of e.s.d.'s in distances, angles and torsion angles; correlations between e.s.d.'s in cell parameters are only used when they are defined by crystal symmetry. An approximate (isotropic) treatment of cell e.s.d.'s is used for estimating e.s.d.'s involving l.s. planes.
Refinement. Refinement of *F*^2^ against ALL reflections. The weighted *R*-factor *wR* and goodness of fit *S* are based on *F*^2^, conventional *R*-factors *R* are based on *F*, with *F* set to zero for negative *F*^2^. The threshold expression of *F*^2^ > σ(*F*^2^) is used only for calculating *R*-factors(gt) *etc*. and is not relevant to the choice of reflections for refinement. *R*-factors based on *F*^2^ are statistically about twice as large as those based on *F*, and *R*- factors based on ALL data will be even larger.

### Fractional atomic coordinates and isotropic or equivalent isotropic displacement parameters (Å^2^)

**Table:**

	*x*	*y*	*z*	*U*_iso_*/*U*_eq_	Occ. (<1)
S1	0.08891 (9)	0.24309 (9)	0.39262 (8)	0.0688 (3)	
O1	−0.0316 (7)	0.2731 (8)	0.3422 (6)	0.143 (2)	0.782 (10)
O2	0.1501 (6)	0.1801 (6)	0.2961 (4)	0.123 (2)	0.782 (10)
O3	0.0335 (7)	0.1588 (5)	0.4409 (6)	0.146 (2)	0.782 (10)
O4	0.2181 (6)	0.3518 (4)	0.4819 (6)	0.145 (3)	0.782 (10)
O1'	−0.034 (3)	0.262 (3)	0.320 (3)	0.177 (7)*	0.218 (10)
O2'	0.033 (3)	0.1150 (12)	0.373 (2)	0.177 (7)*	0.218 (10)
O3'	0.127 (3)	0.3358 (19)	0.5232 (11)	0.177 (7)*	0.218 (10)
O4'	0.231 (2)	0.281 (3)	0.366 (3)	0.177 (7)*	0.218 (10)
O5	−0.0119 (3)	0.2807 (3)	0.8003 (2)	0.0970 (9)	
H5	0.042 (5)	0.344 (3)	0.875 (2)	0.146*	
N1	0.2107 (3)	0.4485 (2)	1.0422 (2)	0.0633 (7)	
N2	0.4816 (3)	0.7105 (2)	1.01850 (19)	0.0505 (6)	
N3	0.4380 (3)	0.7522 (2)	0.9335 (2)	0.0510 (6)	
N4	0.4932 (3)	0.8909 (2)	0.84709 (19)	0.0498 (6)	
H4A	0.3947	0.8512	0.7984	0.060*	
N5	0.5238 (3)	1.0240 (2)	0.7534 (2)	0.0501 (6)	
N6	0.6192 (3)	1.1068 (2)	0.7231 (2)	0.0510 (6)	
N7	0.7420 (3)	1.2708 (2)	0.6334 (2)	0.0594 (6)	
H7	0.7990	1.2619	0.6913	0.071*	
C1	0.2076 (4)	0.3868 (3)	1.1128 (3)	0.0732 (9)	
H1	0.1124	0.3195	1.0930	0.088*	
C2	0.3357 (4)	0.4170 (3)	1.2119 (3)	0.0681 (9)	
H2	0.3274	0.3722	1.2587	0.082*	
C3	0.4761 (4)	0.5147 (3)	1.2404 (3)	0.0653 (9)	
H3	0.5660	0.5361	1.3062	0.078*	
C4	0.4840 (3)	0.5811 (3)	1.1715 (2)	0.0552 (7)	
H4	0.5789	0.6482	1.1902	0.066*	
C5	0.3484 (3)	0.5469 (3)	1.0735 (2)	0.0481 (7)	
C6	0.3476 (3)	0.6161 (3)	0.9981 (2)	0.0506 (7)	
C7	0.2185 (3)	0.5979 (3)	0.9010 (3)	0.0622 (8)	
H7A	0.1134	0.5383	0.8700	0.075*	
C8	0.2800 (4)	0.6859 (3)	0.8621 (3)	0.0611 (8)	
H8	0.2247	0.6984	0.7988	0.073*	
C9	0.5506 (3)	0.8563 (3)	0.9304 (2)	0.0477 (6)	
C10	0.7073 (3)	0.9165 (3)	1.0114 (3)	0.0572 (7)	
H10	0.7430	0.8894	1.0690	0.069*	
C11	0.8084 (4)	1.0183 (3)	1.0032 (3)	0.0636 (8)	
H11	0.9147	1.0614	1.0560	0.076*	
C12	0.7519 (3)	1.0566 (3)	0.9164 (3)	0.0581 (7)	
H12	0.8182	1.1247	0.9090	0.070*	
C13	0.5926 (3)	0.9889 (3)	0.8413 (2)	0.0477 (6)	
C14	0.3652 (3)	0.9869 (3)	0.6920 (3)	0.0565 (7)	
H14	0.2790	0.9308	0.6982	0.068*	
C15	0.3582 (3)	1.0485 (3)	0.6199 (3)	0.0584 (7)	
H15	0.2669	1.0431	0.5669	0.070*	
C16	0.5178 (3)	1.1216 (3)	0.6422 (2)	0.0507 (7)	
C17	0.5826 (3)	1.2052 (3)	0.5893 (2)	0.0515 (7)	
C18	0.4928 (4)	1.2218 (3)	0.4983 (3)	0.0623 (8)	
H18	0.3822	1.1775	0.4658	0.075*	
C19	0.5648 (5)	1.3030 (4)	0.4550 (3)	0.0745 (10)	
H19	0.5028	1.3145	0.3941	0.089*	
C20	0.7278 (5)	1.3675 (3)	0.5008 (3)	0.0786 (10)	
H20	0.7773	1.4223	0.4711	0.094*	
C21	0.8167 (4)	1.3495 (3)	0.5917 (3)	0.0729 (9)	
H21	0.9276	1.3917	0.6238	0.087*	
C22	0.0440 (5)	0.1814 (5)	0.7471 (4)	0.1141 (14)	
H22A	0.0433	0.1681	0.6641	0.171*	
H22B	−0.0246	0.1024	0.7417	0.171*	
H22C	0.1511	0.2066	0.7997	0.171*	

### Atomic displacement parameters (Å^2^)

**Table:**

	*U*^11^	*U*^22^	*U*^33^	*U*^12^	*U*^13^	*U*^23^
S1	0.0483 (5)	0.0784 (6)	0.0764 (6)	0.0165 (4)	0.0085 (4)	0.0448 (5)
O1	0.090 (3)	0.216 (6)	0.144 (4)	0.085 (3)	0.009 (3)	0.102 (4)
O2	0.119 (4)	0.146 (5)	0.108 (3)	0.061 (4)	0.046 (3)	0.053 (3)
O3	0.193 (5)	0.137 (4)	0.151 (5)	0.051 (4)	0.087 (4)	0.105 (4)
O4	0.112 (3)	0.080 (3)	0.146 (4)	0.014 (2)	−0.060 (3)	0.025 (3)
O5	0.0841 (17)	0.0708 (18)	0.0880 (18)	0.0114 (15)	−0.0245 (14)	0.0295 (15)
N1	0.0612 (16)	0.0629 (17)	0.0612 (15)	0.0157 (14)	0.0120 (13)	0.0350 (14)
N2	0.0603 (15)	0.0498 (14)	0.0427 (12)	0.0229 (13)	0.0153 (11)	0.0231 (11)
N3	0.0574 (14)	0.0456 (14)	0.0464 (12)	0.0191 (12)	0.0135 (11)	0.0208 (11)
N4	0.0498 (13)	0.0459 (14)	0.0433 (12)	0.0162 (12)	0.0099 (10)	0.0156 (11)
N5	0.0551 (14)	0.0462 (14)	0.0478 (13)	0.0199 (12)	0.0161 (11)	0.0217 (12)
N6	0.0549 (13)	0.0464 (14)	0.0482 (13)	0.0182 (12)	0.0130 (11)	0.0220 (11)
N7	0.0757 (18)	0.0522 (15)	0.0476 (13)	0.0229 (14)	0.0098 (12)	0.0276 (12)
C1	0.075 (2)	0.071 (2)	0.079 (2)	0.0189 (18)	0.0226 (18)	0.048 (2)
C2	0.079 (2)	0.078 (2)	0.0627 (19)	0.035 (2)	0.0237 (18)	0.0445 (19)
C3	0.082 (2)	0.076 (2)	0.0429 (16)	0.044 (2)	0.0162 (16)	0.0251 (17)
C4	0.0577 (17)	0.0513 (18)	0.0465 (15)	0.0196 (15)	0.0112 (14)	0.0177 (14)
C5	0.0535 (16)	0.0457 (17)	0.0437 (14)	0.0205 (14)	0.0167 (13)	0.0187 (13)
C6	0.0558 (17)	0.0440 (16)	0.0458 (15)	0.0196 (14)	0.0134 (13)	0.0170 (13)
C7	0.0514 (17)	0.0538 (19)	0.0665 (18)	0.0087 (15)	0.0031 (15)	0.0301 (16)
C8	0.0600 (19)	0.0566 (19)	0.0597 (18)	0.0196 (16)	0.0044 (15)	0.0303 (16)
C9	0.0535 (16)	0.0453 (16)	0.0424 (14)	0.0215 (14)	0.0165 (13)	0.0173 (13)
C10	0.0591 (18)	0.063 (2)	0.0544 (17)	0.0257 (16)	0.0172 (15)	0.0314 (16)
C11	0.0526 (17)	0.073 (2)	0.0581 (18)	0.0192 (17)	0.0113 (14)	0.0305 (17)
C12	0.0539 (17)	0.0571 (19)	0.0592 (17)	0.0154 (15)	0.0172 (15)	0.0287 (16)
C13	0.0545 (16)	0.0451 (17)	0.0427 (14)	0.0208 (14)	0.0166 (13)	0.0188 (13)
C14	0.0522 (17)	0.0539 (18)	0.0522 (16)	0.0184 (15)	0.0122 (14)	0.0185 (15)
C15	0.0560 (18)	0.0578 (19)	0.0530 (16)	0.0247 (16)	0.0103 (14)	0.0202 (15)
C16	0.0635 (18)	0.0450 (17)	0.0409 (14)	0.0263 (15)	0.0137 (13)	0.0160 (13)
C17	0.0630 (19)	0.0444 (16)	0.0431 (15)	0.0258 (15)	0.0129 (14)	0.0157 (13)
C18	0.076 (2)	0.068 (2)	0.0525 (16)	0.0421 (18)	0.0160 (15)	0.0295 (17)
C19	0.108 (3)	0.080 (2)	0.0595 (19)	0.057 (2)	0.028 (2)	0.0413 (19)
C20	0.119 (3)	0.068 (2)	0.066 (2)	0.042 (2)	0.036 (2)	0.041 (2)
C21	0.085 (2)	0.060 (2)	0.0634 (19)	0.0145 (19)	0.0183 (18)	0.0320 (18)
C22	0.105 (3)	0.122 (4)	0.106 (3)	0.047 (3)	0.019 (3)	0.051 (3)

### Geometric parameters (Å, º)

**Table:**

S1—O4	1.365 (3)	C3—H3	0.9300
S1—O1	1.378 (3)	C4—C5	1.387 (4)
S1—O3	1.396 (3)	C4—H4	0.9300
S1—O2'	1.400 (9)	C5—C6	1.469 (4)
S1—O4'	1.403 (9)	C6—C7	1.411 (4)
S1—O1'	1.432 (9)	C7—C8	1.359 (4)
S1—O2	1.448 (4)	C7—H7A	0.9300
S1—O3'	1.451 (9)	C8—H8	0.9300
O5—C22	1.422 (5)	C9—C10	1.382 (4)
O5—H5	0.863 (11)	C10—C11	1.378 (4)
N1—C1	1.342 (4)	C10—H10	0.9300
N1—C5	1.344 (3)	C11—C12	1.385 (4)
N2—C6	1.334 (3)	C11—H11	0.9300
N2—N3	1.363 (3)	C12—C13	1.381 (4)
N3—C8	1.362 (4)	C12—H12	0.9300
N3—C9	1.415 (3)	C14—C15	1.362 (4)
N4—C9	1.321 (3)	C14—H14	0.9300
N4—C13	1.321 (3)	C15—C16	1.402 (4)
N4—H4A	0.8600	C15—H15	0.9300
N5—N6	1.357 (3)	C16—C17	1.458 (4)
N5—C14	1.366 (3)	C17—C18	1.372 (4)
N5—C13	1.410 (3)	C18—C19	1.366 (5)
N6—C16	1.329 (3)	C18—H18	0.9300
N7—C21	1.339 (4)	C19—C20	1.370 (5)
N7—C17	1.343 (4)	C19—H19	0.9300
N7—H7	0.8600	C20—C21	1.377 (4)
C1—C2	1.367 (4)	C20—H20	0.9300
C1—H1	0.9300	C21—H21	0.9300
C2—C3	1.364 (4)	C22—H22A	0.9600
C2—H2	0.9300	C22—H22B	0.9600
C3—C4	1.372 (4)	C22—H22C	0.9600
			
O4—S1—O1	111.7 (4)	N1—C5—C6	116.3 (2)
O4—S1—O3	111.2 (3)	C4—C5—C6	121.8 (3)
O1—S1—O3	111.2 (4)	N2—C6—C7	111.2 (2)
O4—S1—O2'	132.2 (10)	N2—C6—C5	120.1 (2)
O1—S1—O2'	112.3 (12)	C7—C6—C5	128.6 (3)
O3—S1—O2'	33.1 (10)	C8—C7—C6	105.5 (3)
O4—S1—O4'	60.1 (10)	C8—C7—H7A	127.2
O1—S1—O4'	116.2 (10)	C6—C7—H7A	127.2
O3—S1—O4'	131.3 (9)	C7—C8—N3	106.9 (2)
O2'—S1—O4'	113.6 (11)	C7—C8—H8	126.6
O4—S1—O1'	116.8 (13)	N3—C8—H8	126.6
O1—S1—O1'	9.8 (16)	N4—C9—C10	124.0 (3)
O3—S1—O1'	114.6 (14)	N4—C9—N3	114.9 (2)
O2'—S1—O1'	109.6 (12)	C10—C9—N3	121.0 (2)
O4'—S1—O1'	110.6 (13)	C11—C10—C9	117.2 (3)
O4—S1—O2	104.5 (3)	C11—C10—H10	121.4
O1—S1—O2	110.1 (3)	C9—C10—H10	121.4
O3—S1—O2	107.8 (3)	C10—C11—C12	120.2 (3)
O2'—S1—O2	77.0 (10)	C10—C11—H11	119.9
O4'—S1—O2	45.5 (11)	C12—C11—H11	119.9
O1'—S1—O2	100.4 (13)	C13—C12—C11	116.9 (3)
O4—S1—O3'	44.6 (9)	C13—C12—H12	121.5
O1—S1—O3'	96.3 (11)	C11—C12—H12	121.5
O3—S1—O3'	79.9 (9)	N4—C13—C12	124.2 (2)
O2'—S1—O3'	112.3 (11)	N4—C13—N5	115.0 (2)
O4'—S1—O3'	104.6 (11)	C12—C13—N5	120.7 (3)
O1'—S1—O3'	105.8 (12)	C15—C14—N5	106.4 (3)
O2—S1—O3'	146.4 (10)	C15—C14—H14	126.8
C22—O5—H5	120 (3)	N5—C14—H14	126.8
C1—N1—C5	117.1 (3)	C14—C15—C16	105.5 (2)
C6—N2—N3	104.5 (2)	C14—C15—H15	127.2
C8—N3—N2	111.9 (2)	C16—C15—H15	127.2
C8—N3—C9	127.6 (2)	N6—C16—C15	111.6 (2)
N2—N3—C9	120.4 (2)	N6—C16—C17	118.6 (3)
C9—N4—C13	117.5 (2)	C15—C16—C17	129.8 (2)
C9—N4—H4A	121.3	N7—C17—C18	117.8 (3)
C13—N4—H4A	121.3	N7—C17—C16	117.4 (2)
N6—N5—C14	112.0 (2)	C18—C17—C16	124.8 (3)
N6—N5—C13	119.9 (2)	C19—C18—C17	120.4 (3)
C14—N5—C13	128.1 (2)	C19—C18—H18	119.8
C16—N6—N5	104.4 (2)	C17—C18—H18	119.8
C21—N7—C17	123.3 (3)	C18—C19—C20	120.4 (3)
C21—N7—H7	118.3	C18—C19—H19	119.8
C17—N7—H7	118.3	C20—C19—H19	119.8
N1—C1—C2	124.0 (3)	C19—C20—C21	118.7 (3)
N1—C1—H1	118.0	C19—C20—H20	120.7
C2—C1—H1	118.0	C21—C20—H20	120.7
C3—C2—C1	118.3 (3)	N7—C21—C20	119.4 (3)
C3—C2—H2	120.9	N7—C21—H21	120.3
C1—C2—H2	120.9	C20—C21—H21	120.3
C2—C3—C4	119.6 (3)	O5—C22—H22A	109.5
C2—C3—H3	120.2	O5—C22—H22B	109.5
C4—C3—H3	120.2	H22A—C22—H22B	109.5
C3—C4—C5	119.0 (3)	O5—C22—H22C	109.5
C3—C4—H4	120.5	H22A—C22—H22C	109.5
C5—C4—H4	120.5	H22B—C22—H22C	109.5
N1—C5—C4	121.9 (2)		

### Hydrogen-bond geometry (Å, º)

**Table:**

*D*—H···*A*	*D*—H	H···*A*	*D*···*A*	*D*—H···*A*
N7—H7···N6	0.86 (1)	2.35 (1)	2.713 (10)	106 (1)
O5—H5···N1	0.86 (3)	1.97 (3)	2.79 (3)	159 (4)
N7—H7···O5^i^	0.86 (1)	1.88 (1)	2.690 (10)	156 (1)

Symmetry code: (i) *x*+1, *y*+1, *z*.
